# The evolution and social cost of herding mentality promote cooperation

**DOI:** 10.1016/j.isci.2023.107927

**Published:** 2023-09-15

**Authors:** Manuel Chica, William Rand, Francisco C. Santos

**Affiliations:** 1 Andalusian Research Institute DaSCI “Data Science and Computational Intelligence”, University of Granada, 18071 Granada, Spain; 2 School of Electrical Engineering and Computing, The University of Newcastle, Callaghan, NSW 2308, Australia; 3 INESC-ID & Instituto Superior Técnico, Universidade de Lisboa, 2744-016 Porto Salvo, Portugal; 4 Poole College of Management, North Carolina State University, Raleigh, NC 27695, USA

**Keywords:** Psychological evolution, Psychology, Sociology

## Abstract

Herding behavior has a social cost for individuals not following the herd, influencing human decision-making. This work proposes including a social cost derived from herding mentality into the payoffs of pairwise game interactions. We introduce a co-evolutionary asymmetric model with four individual strategies (cooperation vs. defection and herding vs. non-herding) to understand the co-emergence of herding behavior and cooperation. Computational experiments show how including herding costs promotes cooperation by increasing the parameter space under which cooperation persists. Results demonstrate a synergistic relationship between the emergence of cooperation and herding mentality: the highest cooperation is achieved when the herding mentality also achieves its highest level. Finally, we study different herding social costs and its relationship to cooperation and herding evolution. This study points to new social mechanisms, related to conformity-driven imitation behavior, that help to understand how and why cooperation prevails in human groups.

## Highlights

• Herding behavior results in a social cost when behaving differently than the crowd
• Social cost is included in the individuals’ payoffs for a pairwise two-player game
• Herding-based social costs promote cooperation in structured populations
• The herding population co-evolves with cooperation, helping cooperators to prevail

## Graphical abstract

**Figure:**
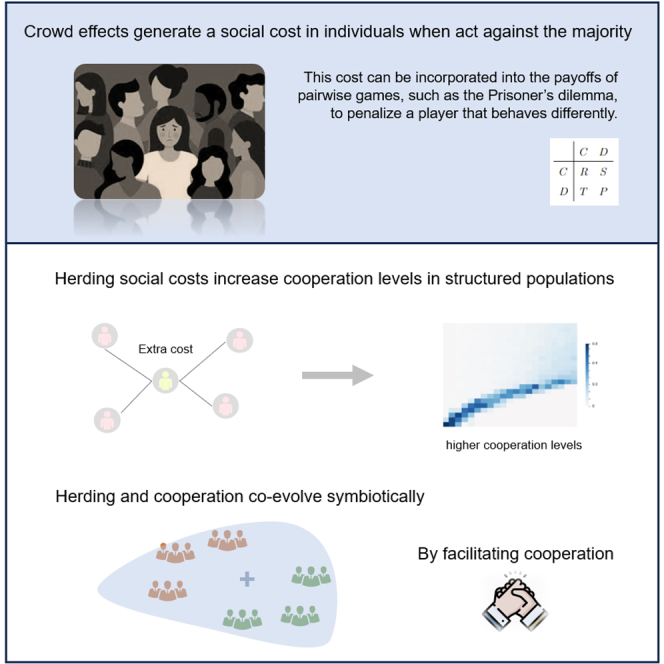

## Introduction

Herding does not come about because a central actor tells the agents to herd, but rather it is an emergent phenomenon of many local decisions, wherein the beliefs and thoughts of individuals become aligned.^1^ Herding has been studied since Adam Smith first pointed out that people often imagine themselves in the shoes of others when making decisions,^2^ but it is only recently that researchers have had the proper tools of social network analysis to help understand how herding decisions move between individuals across dyads.^3^ Herding is a form of social contagion, where one individual adopts the views of another, primarily because it increases their confidence in a decision they were making.^1^ Also, herding is related to conformity, an important behavior in humans’ social learning, being a tendency to act as the majority of the individuals do.^4^^,^^5^ Herd mentality can have a powerful influence on people’s behavior. In some cases, it can lead to positive outcomes, such as when a group comes together to achieve a common goal. But it can also have negative consequences, such as when people conform to harmful stereotypes or engage in risky behavior. This important behavior has been studied in finance and stocks markets,^6^^,^^7^ crypto-currency markets,^8^ online reviews in marketing,^9^ organization and management,^10^ and COVID-19 pandemic effects.^11^

The cost of herding occurs when someone decides to make the opposite decision of the rest of the herd. In general, people feel more comfortable when they follow the opinions and actions of the majority. For instance, recent human experiments revealed that individuals would more likely follow the group’s gaze when more people are looking at them.^12^ Thus, “fighting the herd” creates social costs, since individuals who disagree with the majority are labeled “non-conformers” and do not receive the same social rewards as those who “go with the flow”.^13^ Individuals who adopt a herding mentality suffer consequences when they decide to fight the herd. It is important to realize that these costs are only incurred, if the actor has adopted a herd mentality. If they do not care about the herd, then they do not suffer social costs.

Many herding examples are related to cooperation, where individuals have made the decision to work together as a social group. As a result, it is natural to ask whether herding mentality and the evolution of cooperation are related. The first model-based studies were done by Axelrod,^14^ and Nowak combined the work of Axelrod with modern evolutionary approach to construct evolutionary games that helped explore the mechanisms by which cooperation could persist.^15^^,^^16^^,^^17^ But the evolution of cooperation in biological systems and human groups is an active area of study.^18^^,^^19^^,^^20^ Several mechanisms, such as altruism, the role of kinship relations, selection mechanisms on the group level, and others, have been proposed to explain the robust evolution of cooperation.^16^ Cooperation in humans and societies has been formulated in the framework of evolutionary game theory, resulting in the development of a series of pairwise games to explore cooperation.^21^ Because these pairwise games are highly dependent on the interaction structure, the influence of the network of contacts or social networks has been studied,^22^^,^^23^ and in this study, we will also examine agents involved in pairwise games on networks.

In evolutionary social dilemma studies, the term “conformity” is often used in a related context to herding and was first introduced by Szolnoki and Perc.^5^^,^^24^ Conformity is defined as choosing the most frequent strategy observed by the player, instead of being guided by maximizing their personal payoffs. In these models, a fraction of the population is considered as conformists and their update dynamics are different from the traditional one (i.e., a payoffs-driven update rule). Szolnoki and Perc found that cooperation can be promoted by an appropriate proportion of conformists in the population^5^ and they also confirmed that conformity increases cooperation by enhancing network reciprocity. Other recent studies related to conformity have examined additional behavioral aspects. For instance, the study of Zhang et al.^25^ included, in the same model, conformity-driven (i.e., following the majority), aspiration-driven (i.e., comparing their performance with their aspirations), and payoff-driven agents (i.e., the traditional game where agents only concern about their payoffs). Other authors proposed a model with traditional and rational conformist players, where they can follow their neighbors when their payoffs have decreased in comparison with previous steps.^26^ Quan et al. studied conformity in public good games^4^ and Pi et al. discussed a multi-game model where there are two payoff matrices and two types of players, profiteers (payoffs-driven) and conformists.^27^

The main motivation here is to study the impact of psychological costs of herding in the population dynamics and their levels of cooperation. Thus, we propose the inclusion of a psychological herding cost in a pairwise evolutionary game as a mechanism to explain the evolution of cooperation.^22^ In general, evolutionary theory studies on conformity are able to model different imitation mechanisms in the update rule (e.g., adopting the strategy of the majority of the contacts). In our model, herding behavior is intrinsic to the game because a psychological cost is included in their payoffs (i.e., there is an explicit integration of the herding cost to the payoffs), while the update rule is common for all the players. This cost is calculated from the ratio of connected players behaving differently, i.e., if more connected players uses different strategies then there is less herding, and, as a result, the players acting differently from the herd suffer a cost. In the case of a game with two strategies (either defecting or cooperating), this cost comes from the ratio of connected players adopting the opposite strategy.

Additionally, this work proposes a novel game to understand the evolution of the herding mentality. To achieve this goal, we define a co-evolutionary asymmetric game^28^ where the learning of an additional strategy (i.e., to have a herding mentality or not) is combined with the traditional generalized pairwise game. The key feature of our co-evolutionary game is that players may adopt not only strategies from a neighbor but also a different payoff structure, i.e., herding or not. Thus, the proposed co-evolutionary game features four different strategies ($HC,HD,\tilde{H}C,\tilde{H}D$) where players can either cooperate or defect, with or without a herding mentality and the strategies can be learned in a co-evolutionary context. The main difference between this game and the conformity models is that herding is treated as a social cost in the payoff matrix while the imitation process is equal for all the players. Players with a herding mentality will add the psychological cost when observing the behavior of their direct contacts. Through this new evolutionary game, the evolution of the players having a herding mentality can be observed together with the evolution of cooperation in an asymmetric generalized pairwise game.

## Results

In this section, we examine the dynamics of herding costs, the co-evolutionary process of both herding and cooperation, and the implication of different values for the social herding cost. See the Method details section for more information about the payoffs matrix, evolutionary processes, and simulation setup. In a nutshell, the inclusion of a herding cost modifies the payoff matrix of a pairwise evolutionary game and is applied to the structured population by evaluating the players in the local neighborhood who behave differently (i.e., counting different behaviors in the set of direct contacts of the social network).

By using Monte Carlo (MC) agent-based simulations,^29^^,^^30^ we run the evolutionary model for both the generalized pairwise game with herding costs and a co-evolutionary game with four strategies (i.e., cooperator with herding HC, cooperator without herding $\tilde{H}C$, defector with herding HD, and defector without herding $\tilde{H}D$). All the results are obtained for regular lattice of size 70×70 and scale-free (SF) network of 5,000 nodes, with both networks having same average degree of 4 nodes. Under different settings, results are obtained by averaging 30 MC realizations and by modifying the only two *S* and *T* parameters of the generalized pairwise game as well as the social costs weights of the herding behavior.

### Influence of herding cost in pairwise game

**Figure 1:**
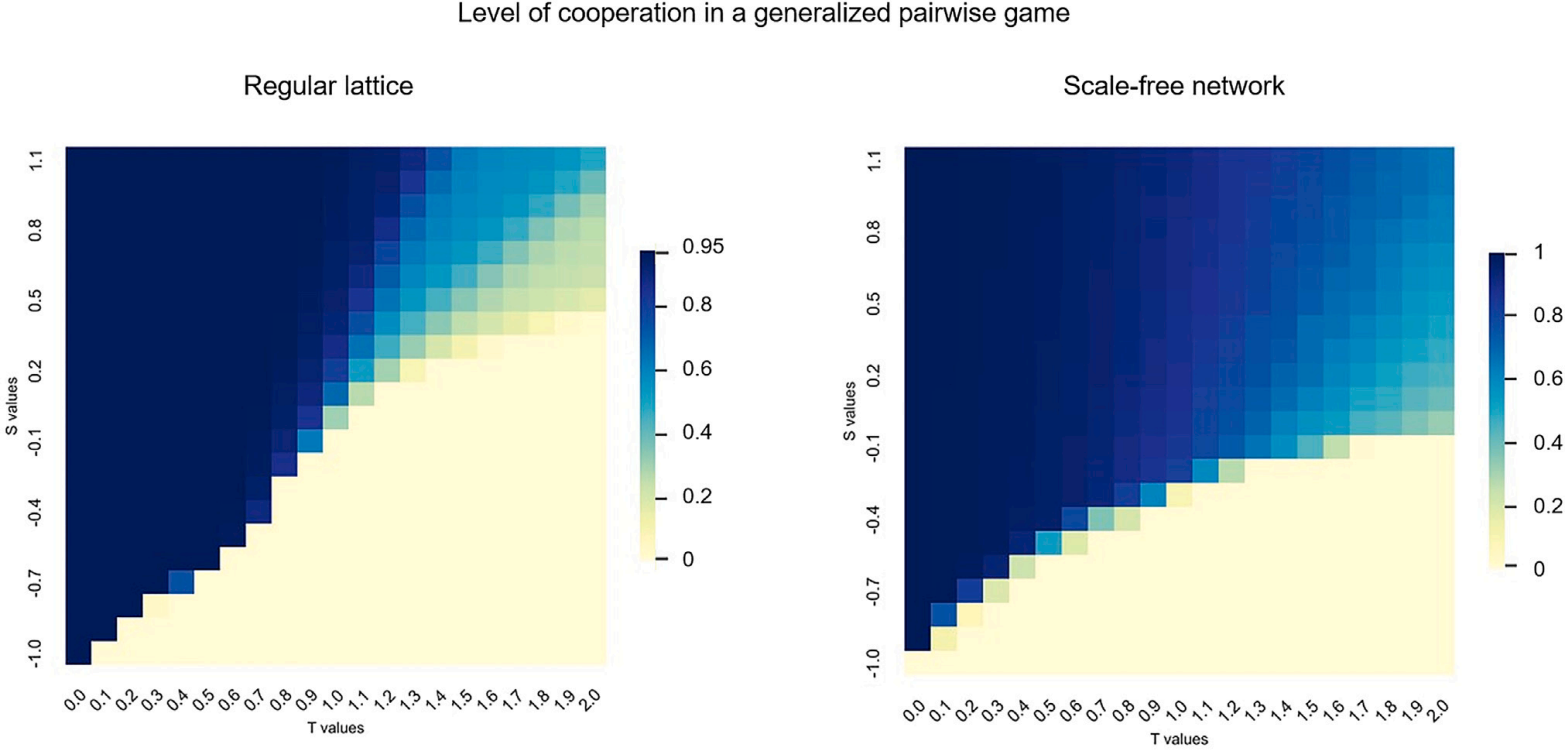
Cooperation levels in generalized pairwise game on *S* and *T* Left plot shows cooperation for a regular lattice and right plot for SF network under different values of *S* and *T*.

**Figure 2:**
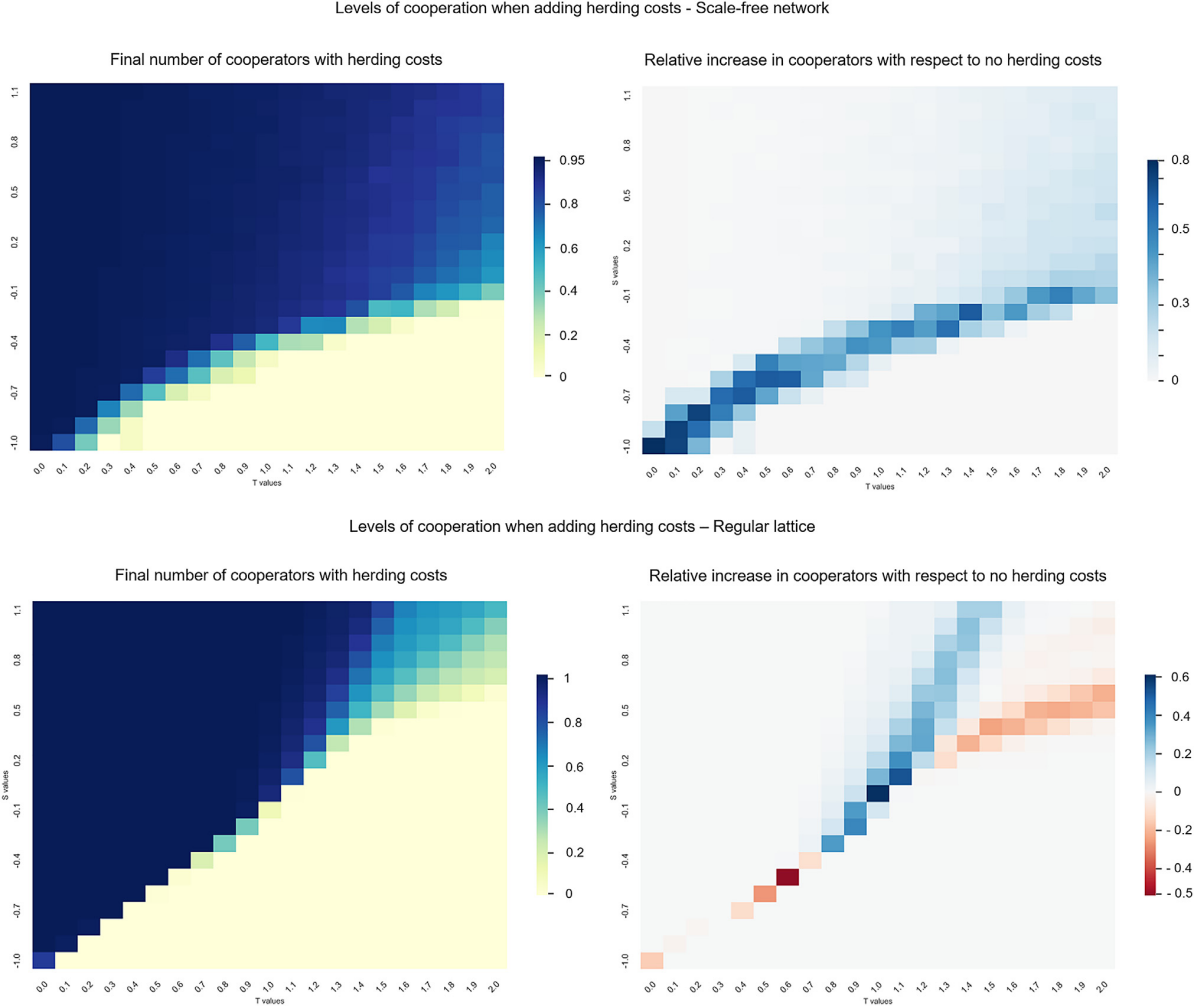
Final levels of cooperation in a lattice and SF when introducing herding costs Left and right plots show, respectively, the absolute cooperation levels and increase in cooperation with respect to the generalized pairwise game for an SA on *S* and *T* when including herding costs in the game. Top plots are for SF network and bottom plots for regular lattice. An important increase in cooperation for the SF is observed. When in a regular lattice, there is an area where we can see an increase of cooperation but also a slighter decrease below the diagonal of the plot.

In the generalized pairwise game, the population of players ends in a state of full defection, full cooperation, or a co-existence stationary state depending on the values of parameters *S* and *T*. Figure 1 illustrates final levels of cooperation for two networked populations, a regular lattice and an SF network, when varying the values of *S* and *T*. Figure 2 shows a sensitivity analysis surface on the main parameters *S* and *T* of the pairwise game for the regular lattice and SF networks to see the herding cost effects in the game. The values represented in the heatmaps are both the final level of cooperation and relative increases with respect to the baseline output of a pairwise game without considering herding costs (i.e., social influence τ=0).

We see that, both in the lattice and SF, there is an increase in cooperation when adding herding mentality as a social cost. When the game is on the SF network (top plots of Figure 2), there is a shift in the cooperation area of the generalized pairwise two-player game. Apart from this shifting in the transition zone, a high level of cooperation is achieved in a large area behind the transition zone. In the case of a lattice (bottom plots), we see a small decrease in the level of cooperation in the transition zone when *T* and *S* are low. However, in general, the level of cooperation is higher, than the SF network, although differences are not as significant as with the SF network. Thus, including herding costs may promote cooperation, and this effect may be stronger in heterogeneous networks, such as SF.

### Co-evolutionary analysis of cooperation and herding

**Figure 3:**
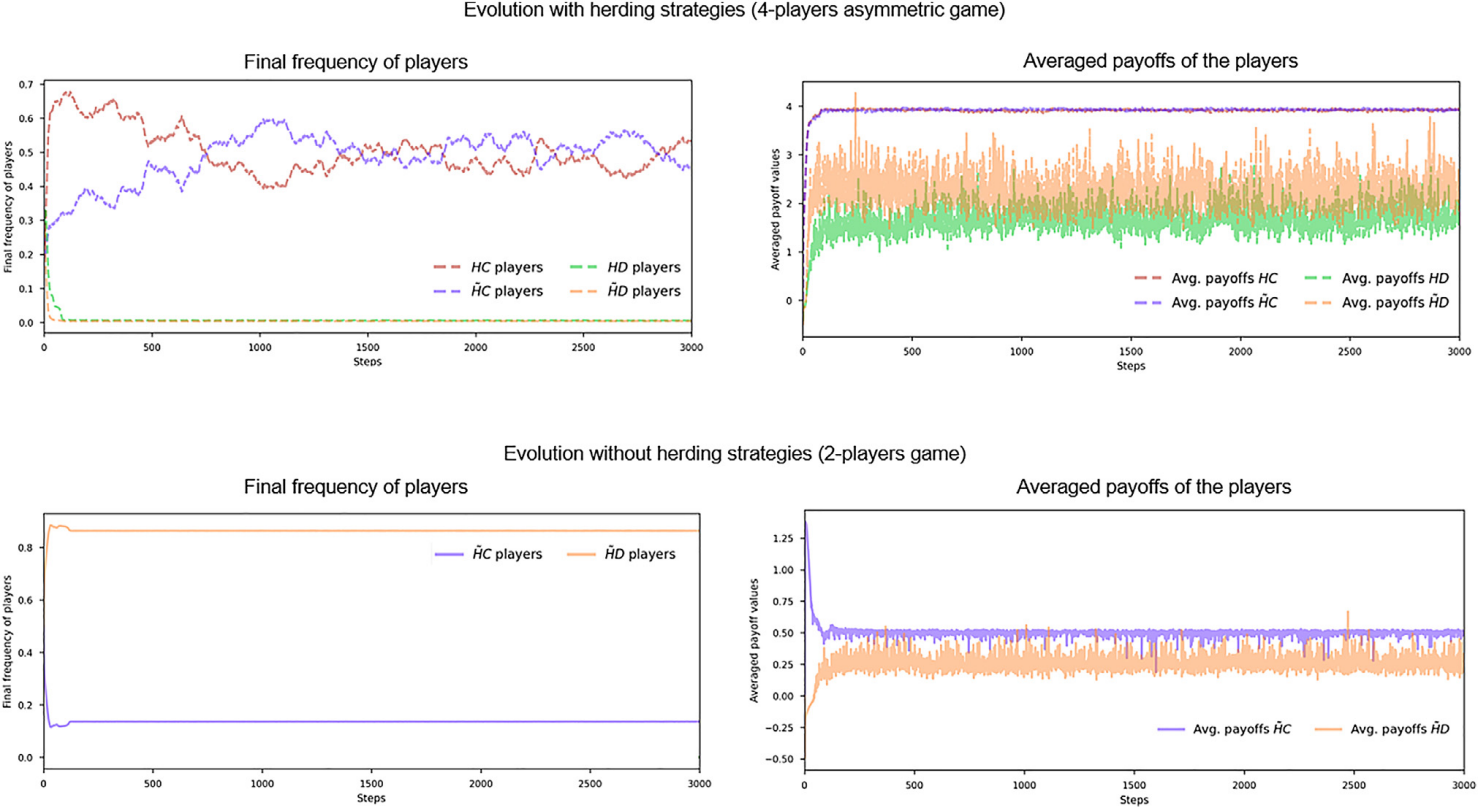
Evolution of strategies and payoffs over time Left plots show evolution of frequency of strategies and right plots do for their averaged payoffs in the four strategies co-evolutionary game (first row) and generalized pairwise game (second row). Settings are S=−1,T=0 on an SF network for 3,000 steps. The introduction of the herding strategy rapidly moves the population to full cooperation until the end of the simulation as both HC and $\tilde{H}C$ payoffs are higher than those of HD and $\tilde{H}D$.

In this experiment, we simulate the four strategies asymmetric game, i.e., players have to choose between cooperating and defecting, and herding or not herding. This results in four potential strategies, i.e., HC, $\tilde{H}C$, HD, and $\tilde{H}D$, where *C* is cooperating, *D* is defecting, *H* is herding, and $\tilde{H}$ is not herding. We initialize the simulation with 25% of players of each strategy and the results are again obtained for a lattice and an SF network. First, we illustrate in Figure 3 how strategies and payoffs evolve for *S* equals to −1 and *T* equals to 0. The figure shows a pair of plots for the evolution of the four strategies game^31^ and the averaged payoffs of each strategy in an SF setting. From the figures, we see how the introduction of strategies with a herding cost rapidly moves the population to full cooperation in a scenario where full detection occurred without herding strategies. The introduction of a herding strategy provides defectors with less incentives to defect and increases the number of actors that are cooperating (both HC and $\tilde{H}C$). A static frequency of cooperators is never reached in the four co-evolutionary game as there is always a transfer between cooperating players having HC and $\tilde{H}C$ strategies.

**Figure 4:**
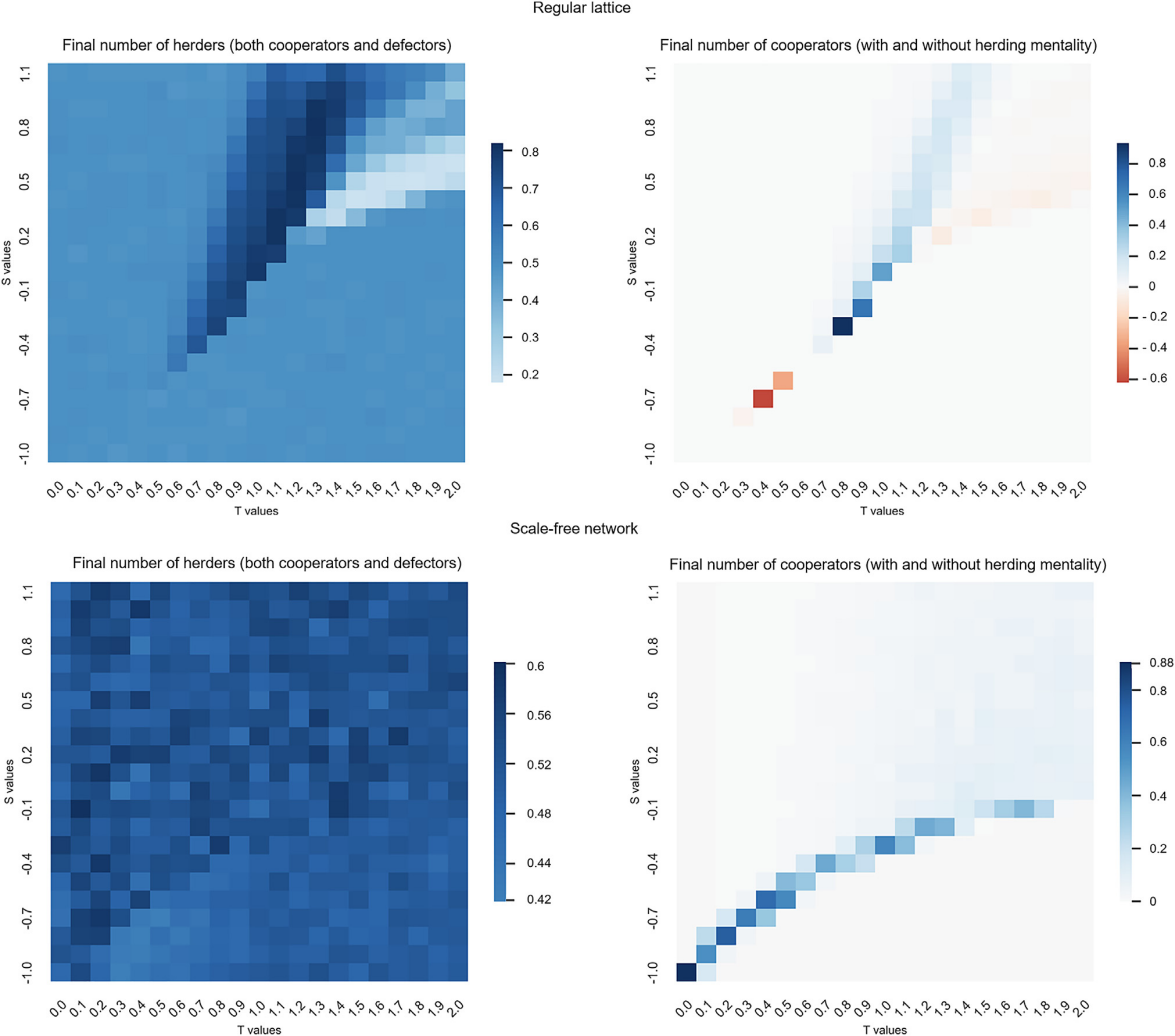
Increase in herding and cooperation levels for lattice and SF Increase in final frequency of players adopting herding and cooperation with respect to the baseline generalized pairwise game for an SA on *S* and *T*. The first row is obtained in a regular lattice and the second row is in an SF network.

We expand the analysis on the values of *S* and *T* and the type of network by running a sensitivity analysis on *S* and *T*. Figure 4 shows pairs of heatmaps of the increase, above the baseline pairwise game, in the final number of herders (players with either HC or HD strategies) and cooperators (players with either $\tilde{H}C$ or HC strategies) for a regular lattice and SF network. From the results of the sensitivity analysis on *S* and *T* on both topologies, we can observe the following dynamics:

• The herding strategy results in more cooperation across the whole space of the generalized pairwise game for both network topologies.
• There is a co-evolution of cooperation and herding. In those areas where cooperation is higher, the prevalence of the herding strategy is also greater.
• We find differences among homogeneous (lattice) and heterogeneous (SF) networks. In SF networks, we find the final number of players having herding behavior is similar for all the game’s parameters and is stable (around 50% of the population) and always high. Nevertheless, there is a drop in herding when full defection happens in these games (low values of *S*).
• However, under the lattice setting (first row of Figure 4), there is an increase in the number of players utilizing the herding strategy when there is an increase of cooperation. The opposite occurs when herding decreases and then defection emerges. Thus, we can see a synergistic effect between herding and cooperation. When one increases, the other increases as well. Heterogeneity in SF networks hide this effect, although higher number of herders are present when there is almost full cooperation.
• Nevertheless, herding players (either HD or $\tilde{H}D$) always coexist with non-herding players, independently from the pair of values for parameters S,T. Even when full defection or full cooperation is easy to achieve, herding players prevail although they do not have a significant impact on the final global output of the population.

### Different influence weights of the social cost

**Figure 5:**
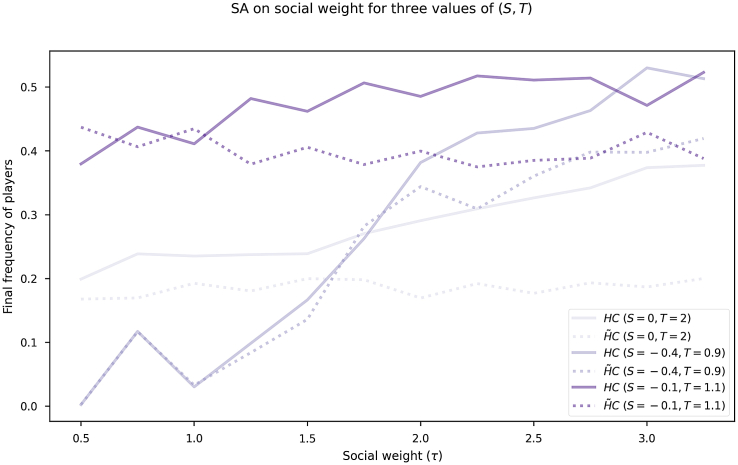
Social weight impact on co-evolutionary herding and cooperation levels Sensitivity analysis on social weight τ for the herding behavior for three pairs of *S* and *T*: (−0.4,0.9), (0,2) and (−0.1,1). The model includes the co-evolution of herding mentality and cooperation (SF network setting).

In order to dig into the practical impact of the model, we evaluate in this set of experiments how different weights of social cost τ influence the evolution of the herding mentality and cooperation levels. Parameter τ controls the weight of the influence of pro-sociality as a herding cost. We run a sensitivity analysis on τ from 0.5 to 3 for the SF setting and the asymmetric game of four strategies to observe the evolutionary dynamics of herding and cooperation in the population. Figure 5 shows three pairs of lines (a solid one for HC and a dashed one for $\tilde{H}C$) representing three values of *S* and *T* ((−0.4,0.9), (0,2), and (−0.1,1.1)) in a networked population having an SF network topology.

From Figure 5, we see how the number of players having both herding and cooperation (strategy HC) always increases with a higher weight τ, as expected. Herder cooperators $\tilde{H}C$ are normally correlated to the cooperators without a herding mentality and are increasing with higher values of τ. Another interesting insight when analyzing this experiment is the fact that the positive difference between the number of HC and $\tilde{H}C$ players increases when increasing the social weight τ. With low values of τ, cooperators and herding cooperators are similar under the three scenarios. However, difference between both strategies is greater when τ≥2. This means that, when achieving a sufficiently high value of τ, the final frequency of herders $\tilde{H}C$ does not increase; but high social cost τ helps to increase the frequency of non-herding cooperators HC.

**Figure 6:**
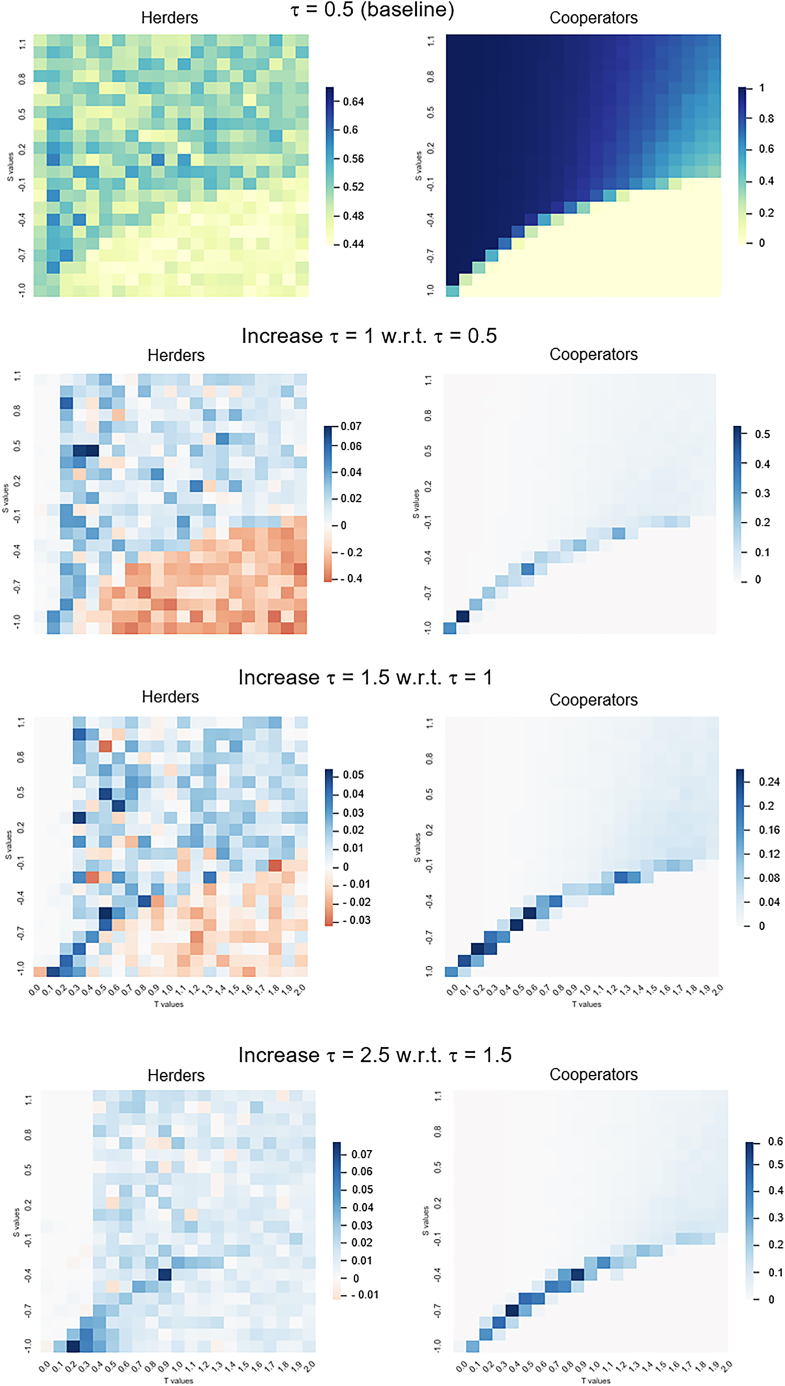
Increase in herding social costs boosts cooperation Rows of heatmaps when running an SA on (S,T) and increasing the values of τ ({0.5,1,1.5,2.5}) in an SF setting. We plot the final number of herders ($\tilde{H}C+\tilde{H}D$) in the first column and final number of cooperators ($HC+\tilde{H}C$) in the second column. Higher social weight values increase cooperation for the whole map of values of the pairwise game.).

Additionally, we run sensitivity analysis on S,T for values of τ={0.5,1,1.5,2.5}. Heatmaps of Figure 6 show the variations in herders ($\tilde{H}C+\tilde{H}D$) and cooperators ($HC+\tilde{H}C$) at the end of the simulation when increasing τ (from τ=0.5 in the first row to τ=2.5 in the last row). As expected, when increasing τ, the full cooperation area becomes wider. Even with a low value (τ=0.5), positive changes are obtained with respect to the case of the generalized pairwise game without herding costs. A general trend when increasing τ (from the second to the fourth row of Figure 6) is a shift in the cooperation transition zone for cooperators. Also, the number of herders is decreasing in those areas when full defection happens when increasing the social weight. The opposite occurs above the transition zone (i.e., full cooperation): higher social weights means a higher number of herders. Finally, another remark is the absence of changes in the herders (first column) when increasing social weight τ for low values of *T* (left part of the heatmaps). In fact, this area of no changes is shifting to the right (higher values of *T*) when increasing the social weight.

## Discussion

The experiments included the comparison of the pairwise game dynamics having herding costs in the local neighborhood in both homogeneous and heterogeneous structured populations (specifically, regular lattice and SF networks). These experiments showed that herding mentality robustly emerges in the four-strategy game through synergistic effects among herding and cooperation. Additionally, we also explored how the herding subpopulation evolves in the asymmetric game for a regular lattice and SF network and assigned different social weights for the psychological cost of having a herding mentality, impacting the final number of herders and cooperators.

Many mechanisms that have been explored to ensure robust cooperation require the specification of an additional agent feature, e.g., tags,^32^ for cooperation to evolve. But in this paper, we hypothesize that the natural social costs associated with herding are sufficient to explain the robust evolution of cooperation. Additionally, previous work has shown that herding can effectively induce cooperation,^33^^,^^34^ but that was in a world where agents were forced to herd, or conform.^5^ This paper illustrates how herding and cooperation can simultaneously evolve increasing the overall level of cooperation and is focused on the psychological costs of herding, explicitly included in the payoffs matrix. This proposed mechanism can be seen as similar to conformity-driven strategies in games^5^^,^^24^^,^^25^ but while keeping the same evolutionary mechanisms. Concretely, Zhang et al. found that the alliances of conformity-driven and aspiration-driven cooperators can greatly boost cooperation in a rather large parameter space.^25^

However, why do cooperation and herding mutually reinforce each other? The intuition behind the obtained results is that defectors, in traditional pairwise games such as Prisoner’s dilemma, take advantage of being surrounded by cooperators. However, defectors are not able to enjoy the profits of being free riders if they adopt the herding mentality. So in cases where everyone is herding, cooperation will prevail. In cases where there is a mixture of strategies near each other, with some individuals herding and some others not herding, individuals who defect against each other are not going to do well because defectors do not score well when playing with each other, and groups of cooperating but not herding individuals can be invaded by defecting strategies. Thus, having a herding mentality has a positive overall effect and can explain why cooperation prevails even without altruism, kin selection, tags, and reciprocity.^17^

If the hypothesis that herding and cooperation naturally co-evolve is correct, that could have important implications for social systems. If we want to reduce herding, for instance, to decrease stock bubbles,^35^ then we could take actions to reduce herding costs such as decreasing the social repercussions of failure making it easier for people to go against the herd.^36^ Or if we want to increase herding, for instance, to encourage the adoption of vaccines, for instance, we could increase the social cost of violating the norm.^37^ Of course, increasing and decreasing the social costs is not easy, since it requires a societal shift, but interventions have worked in the past to alter social norms, such as programs to promote the wearing of bicycle helmets.^38^

Computationally speaking, the idea behind the proposed model is novel. Previous models incorporated herding behavior by modifying the evolutionary mechanisms with additional information^34^ or different evolutionary processes.^5^ In this model, endogenous information for the players are used instead (i.e., the fraction of different strategies played by direct contacts). This information about peers’ actions is used to define the social cost within the payoffs for both 2- and 4-player games. In other words, herding mentality is part of the game itself rather than a property of the players.

More importantly, when herding behavior is an additional strategy and we convert the generalized pairwise game into an asymmetric game with four strategies, one can observe that there are synergistic effects between cooperation and herding costs, as in other co-evolutionary 4-strategy games.^39^ When more cooperation is obtained, a higher number of herders are also observed in the population. Additionally, results show that the herding mentality always prevails in the population independently from the parameters of the game. Finally, we explored the intensity of herding cost. When fighting the herd has a higher cost, the number of players with both cooperation and herding increases, while the fraction of players using the defection strategy decreases.

### Limitations of the study

The current study focuses on exploring the social effects of herding behavior by incorporating a herding social cost and additional strategies for interacting individuals or players. However, this work could be further extended. The social costs from the herding mentality could be revised to other social mechanisms such as emotional effects, e.g., getting mad at a former defector, and reputation, e.g., players gain a reputation as cooperating or defecting, both of which can affect the way individuals play the game.^40^ This could involve changing the way that the herding cost is calculated, and potentially could be combined with the work on tagging and cooperation.^32^ Another future extension of the game would be to allow for a two-step process, in which players might first reflexively herd, and then decide if herding is strategic for them. This is similar to patterns seen in empirical work examining social influence in groups.^12^ Finally, the model could be extended to not just take the social consequences (or costs) of herding into account but also the information acquisition benefits, i.e., those that herd do not have to spend as much time gathering information about their decisions.^37^

Additionally, future studies could explore the implementation of herding behavior in other social dilemmas. For example, future work could explore the influence of herding social costs in public good games,^4^ such as collective-risk dilemmas.^41^ In those dilemmas, groups of players need to establish institutions to preserve the common good and control “free riders”.^42^^,^^43^ These institutions are usually diverse (e.g., local and global institutions) and herding behavior could be incorporated into the institutions’ establishment to see how groups, initially without institutions, follow the “herd” of other groups.

## STAR★Methods

### Key resources table

**Table:** 

REAGENT or RESOURCE	SOURCE	IDENTIFIER
**Software and algorithms**		

Java v11	https://www.oracle.com/pt/java/technologies/javase	N/A
Mason v20	https://cs.gmu.edu/eclab/projects/mason/	N/A
GraphStream v2.0	https://graphstream-project.org/	N/A
NumPy v1.25.1	https://github.com/numpy/numpy	N/A
Matplotlib v3.5.1	https://matplotlib.org	N/A
Pandas v1.4.3	https://scipy.org	N/A
Seaborn v0.11.2	https://seaborn.pydata.org	N/A

### Resource availability

#### Lead contact

Further information and requests for resources should be directed to and will be fulfilled by the lead contact, Manuel Chica (manuelchica@ugr.es).

#### Materials availability

No materials were newly generated for this paper.

### Method details

#### Generalized pairwise game in a population of players

Interactions among individuals can be modeled in terms of two-person games in which both players can either cooperate or defect when interacting with each other.^22^ A cooperation pairwise two-player game is described by a payoff matrix based on four parameters *R* (reward), *S* (sucker’s payoff), *T* (temptation to defect), and *P* (punishment). The payoff matrix in the generalized pairwise game includes different two-strategy games depending on the values of the parameters. These games determine the payoff of every player to either cooperate or defect, and therefore influence the final outcome of the evolutionary game. Mutual cooperation leads to the reward (*R*) whereas mutual defection leads to the punishment (*P*). The other two possibilities occur when one player cooperates and the other defects, for which the associated game payoffs are, the sucker’s payoff (*S*) and temptation (*T*) for the cooperator and the defector, respectively.

The definition of the payoff matrix satisfies R>S and T>P, which encourages the evolution of cooperation in the game. According to Allen and Nowak,^44^ a social dilemma occurs when R>P (mutual cooperation benefits both players) and at least one of the following conditions is met to favor the adoption of defection: $\left(D_{1}\right):T>R$, $\left(D_{2}\right):P>S$, or $\left(D_{3}\right):T>S$. Below appears a payoffs matrix for a generalized pairwise game:

**Table:** 

	*C*	*D*
*C*	*R*	*S*
*D*	*T*	*P*

Without loss of generality we can normalize mutual cooperation *R* to 1 and mutual defection *P* to 0.^22^ Therefore, we normalize the advantage of mutual cooperation over mutual defection in all the games. Also, the game is left with two main parameters: *T* and *S*, which are sufficient to characterize the main pairwise games.^22^ A simplified payoff matrix by just including these two parameters S∈[−1,1] and T∈[0,2] is presented below:

**Table:** 

	*C*	*D*
*C*	1	*S*
*D*	*T*	0

In this simplified version of the payoffs matrix, the snowdrift game (SG) occurs when T>1>S>0 and then, players prefer unilateral defection to mutual cooperation. The stag-hunt (SH) game does when 1>T>0>S and then, players prefer mutual defection to unilateral cooperation. Finally, the prisoner’s dilemma (PD) takes in place when T>1>0>S when both tensions above are incorporated in this dilemma.

Two-person interactions in populations can be modeled by a population *Z* with a finite number of players. Every player *i* can choose its strategy $s_{i}\left(t\right)$ as in a traditional pairwise game at every time step *t*, until reaching a maximum of *T* steps: cooperation (*C*) and defection (*D*). Each player *i* accumulates their payoffs at each time step *t* from all the pairwise interactions in $\Pi_{i}$. The number of game interactions of each player *i* depends on their direct contacts in a network in the case of structured populations. In a well-mixed configuration, a player *i* will interact with all other players, but in this paper we are exploring a more limited number of interactions.

#### Inclusion of a social cost from herding mentality

The psychological costs of a herding mentality $h^{c}$ are directly subtracted from the accumulated payoff value $\Pi_{i}$. This is the cost that players pay for the fraction of their connected players that are utilizing a different strategy, if the player has adopted the herding mentality. Thus, $\Pi_{i}^{\prime }\left(t\right)=\Pi_{i}\left(t\right)-h^{c}\left(t\right)$ for all the players at each time step *t*. This cost $h^{c}$ is calculated as in Equation 1. *C* is the crowd set the focal agent *i* compares with, $r_{i}$ is the ratio of players in *C* having different strategy (behavior) from the *i* player’s strategy, defined by $r_{i}=\frac{1}{\left|C_{i}\right|}\sum_{\forall j\in C_{i}|s_{j}\neq s_{i}}1$. So $r_{i}$ is 0 when all connected players are playing the same strategy and 1 if the focal player is using a different strategy than all connected players. Finally, τ is a positive number measuring the strength of the social weight influence of the herding mentality. If τ=0, the model omits the herding mentality and the generalized pairwise game takes place.

(Equation 1) $$h_{i}^{c}=\tau \frac{\left|C_{i}\right|}{\left\langle k\right\rangle }r_{i}$$

When having a structured population and the comparison is local, the crowd set is $\left|C_{i}\right|={\left\langle k\right\rangle }_{i}$, being ⟨k⟩ is the degree of the player in the network. However, in the absence of a structured population in the model (i.e., when considering a well-mixed setting), each focal agent *i* compares its behavior with the crowd of the whole population. Thus, the size of the crowd set is $\left|C_{i}\right|=Z-1$.

After accumulating the payoffs of all the interactions and discounting the herding cost ($\Pi_{i}^{\prime }$) at time-step *t*, players can update their strategies according to the received payoffs by using the Fermi function as the evolutionary update rule.^45^^,^^46^ The Fermi rule is a stochastic pairwise comparison rule in which strategies that do well, are more likely to be imitated, and spread throughout the population. A focal player *i* can revise its strategy by selecting another player *j* from the set of local contacts in the network. Player *i* will imitate the strategy of *j* with a probability *p* that increases with their payoff difference ($\Pi_{j}^{\prime }-\Pi_{i}^{\prime }$) as in Equation 2:

((Equation 2) $$p=\frac{1}{1+e^{-\beta \left(\Pi_{j}^{\prime }-\Pi_{i}^{\prime }\right)}}\text{.}$$

Free parameter β is the intensity of selection, encoding the chance of mistakes during the imitation process. This means that a player *i* can copy another player’s strategy *j* despite having a lower payoff. The dynamics of the game also includes a mutation function to randomly change the strategies at every time step *t*. A player *i* with just cooperation strategy or playing in the asymmetric game of four strategies can change its strategy at random with a mutation probability μ and imitates the strategy of a local neighbor with probability 1−μ.

#### Herding behavior in an asymmetric co-evolutionary game

In order to study the evolution of herding as an emergence behavior and strategy for the players, we introduce in this section an asymmetric game,^28^ usually also called bimatrix games, where players have four available strategies, coming to be either cooperators or defectors as well as having or not a herding behavior. These four strategies are defined as $s_{i}\left(t\right)=\left\{HC,HD,\tilde{H}C,\tilde{H}D\right\}$. This is a co-evolutionary game where learning strategies compete in the same space.^39^

In this asymmetric co-evolutionary game of four strategies, the update rule is the same for all the players. However, payoffs are updated with the herding cost when evaluating them before applying the update evolutionary function depending on the herding strategy or learning protocol. The base payoffs of each pairwise game are the same as in the generalized game, previously defined. By also using the same Fermi function, focal player *i* having a herding strategy (either HC or HD), will update both its payoff $\Pi_{i}$ and contact’s payoff $\Pi_{j}$ of selected player *j* by $\Pi_{i}^{\prime }=\Pi_{i}-h_{i}^{c}$ and $\Pi_{j}^{\prime }=\Pi_{j}-h_{j}^{c}$.

Therefore, the game updates payoffs with herding costs for focal players who have adopted the herding mentality. As this is the point of view of the focal agent, the agent to be compared with when imitating (*j*) will also have the herding cost discounted. On the contrary, if a player has no herding strategy (either $\tilde{H}C$ or $\tilde{H}D$), it will use the standard payoffs $\Pi_{i}$ and $\Pi_{j}$ when applying the Fermi function.

This cost is added to the payoff for players having a herding mentality *H*. The reason is players with this mentality observe their contacts and the crowd with their perspective and therefore, the payoff depends on the herding mentality of the focal agent but it is not applied to the other player if it has no mentality. This is the reason why the game is asymmetric.

Finally, players with different or equal herding profile can interact and obtain payoffs according to the payoffs’ matrix. In other words, a player who has adopted the herding mentality and one who has not can interact, i.e., from an evolutionary point of view, players with different genotype (i.e., herding behavior) can interact and obtain payoffs from these interactions.^28^ Also, the probability for applying mutation and imitation rules as well as the mutation operator are done as defined in previous Section Inclusion of a social cost from herding mentality but by including the four possible strategies $s_{i}\left(t\right)=\left\{HC,HD,\tilde{H}C,\tilde{H}D\right\}$.

#### Setup and models’ specifications

The EGT experiments^47^ are developed using Monte-Carlo (MC) agent-based simulations,^29^^,^^30^ performed on computer clusters to obtain the stationary states of the model specifications. The model was programmed in the Java programming language and was created specifically for this paper. Evolution proceeds in discrete steps involving the payoffs accumulation for all the groups each player takes part and social imitation through update rules within the whole population or specific network. All of the model results are averaged over 30 independent MC realizations and for a maximum number of $3\times 10^{3}$ synchronous time steps, reaching a stationary stable state and achieving a low deviation of the MC realizations. The presented results were obtained by averaging the last 25% of the simulation time steps in the independent MC realizations.

The mutation (or exploration) probability μ is set to 0.01. We also set β=5 in all the experiments of the study for the Fermi imitation rule. Experiments are carried out using two different network topologies with different heterogeneity levels: scale-free (SF) networks, and regular lattice. The two topologies have similar average degree ⟨k⟩≈4 and density (0.004). Concretely, we use the Barabasi-Albert algorithm^48^ (main parameter of the algorithm m=3) to generate scale-free (SF) networks with an average degree of 4. In order to avoid anomalous network artifacts, we employ different SF networks for the MC runs. We employ a population of size $Z=5\times 10^{3}$ for the SF network and Z=70×70 for the regular lattice in all the experiments. τ equals to 1 for the herding cost $h^{c}$ calculation and then, herding costs are normalized to 1 for the whole experiments unless specified other values.

## Data Availability

• Data generated by the simulations is available upon request. No specific data-sets were used to feed the simulations. • The code used to generate the simulations and the figures is available from the corresponding authors upon request. • Any additional information required to reanalyze the data reported in this paper is available from the lead contact upon request.
